# Interobserver Agreement in Detecting Spectral-Domain Optical Coherence Tomography Features of Diabetic Macular Edema

**DOI:** 10.1371/journal.pone.0126557

**Published:** 2015-05-21

**Authors:** Ling Zhi Heng, Maria Pefianaki, Philip Hykin, Praveen J. Patel

**Affiliations:** NIHR Moorfields Biomedical Research Centre at Moorfields Eye Hospital NHS Foundation Trust and UCL Institute of Ophthalmology, London, United Kingdom; University of Florida, UNITED STATES

## Abstract

**Purpose:**

To evaluate interobserver agreement for the detection of spectral-domain optical coherence tomography (SDOCT) features of diabetic macular edema (DME).

**Method:**

Cross-sectional study in which 2 retinal specialists evaluated SDOCT scans from eyes receiving treatment for DME. Scans from 50 eyes with DME of 39 patients were graded for features of DME including intra-retinal fluid (IRF), diffuse retinal oedema (DRE), hyper-reflective foci (HRF), subretinal fluid (SRF), macular fluid and vitreomacular traction (VMT). Features were graded as present or absent at zones involving the fovea, 1mm from the fovea and the whole scan of 49 line scans. Analysis was performed using cross-tabulations for percentage concordance and kappa values (κ).

**Results:**

In the 2950 line scans analysed, there was an increase in percentage concordance for DRE and HRF when moving from a foveal line scan, 1mm zone and then to a whole scan analysis (88% vs 94% vs 96%) and (88% vs 94% vs 94%) respectively with κ ranging from substantial to almost perfect. Percentage concordance for SRF was 96% at all 3 regions analysed, whilst IRF was 96% at fovea and 98% at higher number of line-scans analysed. Concordance for MF was 100% at fovea and 98% at 1mm zone and whole scan with almost perfect and substantial κ respectively. κ agreement was substantial for VMT at all regions analysed.

**Conclusion:**

We report a high level of interobserver agreement in the detection of SDOCT features of DME. This finding is important as detection of macular fluid is used to guide retreatment with anti-angiogenic agents.

## Introduction

Optical coherence tomography (OCT) imaging has become an essential tool in assessing macular structure. OCT imaging is a rapid, non-invasive imaging modality which uses reflectivity of low coherence light to produce 3 dimensional images of macular structure and is the optical analogue of ultrasound. The technology has undergone rapid development over the past 10 years and the latest spectral-domain OCT (SDOCT) devices are much faster than older time-domain OCT technology permitting more sampling of the macula with reduction in scan times.

The recent and rapid development in retinal imaging has been paralleled by equally impressive improvements in treatments for patients with macular disease. One example of this is in the treatment of diabetic macular edema (DME) where anti-angiogenic agents which block vascular endothelial growth factor (anti-VEGF agents) delivered by intravitreous injection have shown greater efficacy than conventional macular laser in improving vision in eyes with DME.[1,2]

One of the challenges of treatment is in individualising therapy based on signs of DME disease activity. New treatment paradigms therefore rely on using OCT imaging to guide retreatment with anti-VEGF agents after an initial loading phase of treatment when intravitreous injections are given on a regular basis. Assessment of disease activity in DME using OCT imaging relies on using quantitative information about retinal thickness and qualitative information about abnormalities of retinal morphology (typically hyporeflective areas in or below the neurosensory retina suggesting macular fluid) to guide retreatment with anti-VEGF agents. However, changes in macular thickness on OCT can arise from test-retest variability and disagreement over the presence or absence of abnormalities of retinal morphology on OCT imaging can result from inter-observer variability. In previous work we evaluated the repeatability of quantitative SDOCT measures of retinal thickness in patients with DME [3]. In this work we investigate the interobserver variability in interpreting retinal morphological abnormalities on SDOCT images in eyes with DME undergoing treatment. Understanding inter-observer variability is important as disagreements between clinicians in interpreting SDOCT scans in patient receiving anti-VEGF treatment for DME can lead to variability in retreatment decisions, potentially leading to variability in treatment outcomes and variability in the real-world cost-effectiveness of the therapeutic agent. This is also given added importance with the significant costs of delivering care to patients with diabetic macular edema [4] and the prevalence of DME across the world from the Americas through to Asia.[5,6]

## Methodology

All SDOCT imaging was performed using the Spectralis OCT device (Heidelberg Engineering, Heidelberg, Germany), which has a theoretical axial resolution of 3 microns and allows real-time line scan averaging using eye tracking capability (“automatic real-time”; ART mode) to improve the signal-to-noise ratio in line scans.

### Patients

SD-OCT scans acquired using the Spectralis OCT in eyes of consecutive patients with centre involving DME and had undergone previous treatment, either laser or anti-VEGF injections in a 8 month period (from 7 March 2012 to 20 November 2012) as part of a clinical trial (LUCIDATE or OZLASE) in the Clinical Research Facility at Moorfields Eye Hospital were identified by searching the Heidelberg Explorer image database. The participants provided their written consent for the study as part of the consent of the LUCIDATE or OZLASE trial they participated in. The local IRB- Moorfields Eye Hospital Research Management Committee approved this consent procedure. Approval for the collection and analysis of OCT images was obtained from the Research Governance Committee of Moorfields Eye Hospital and by the appropriate Steering Committees of clinical trials where relevant. The research followed the tenets of the Declaration of Helsinki.

Patients underwent imaging performed after visual acuity measurement and pupil dilation with one drop of 2.5% phenylephrine hydrochloride and 1% tropicamide. All patients had given consent to OCT imaging as part of clinical trial involvement.

### Image Acquisition and Scanning Protocol

All OCT scanning was performed by experienced ophthalmic technicians with certification for clinical trials work adhering to a standardized imaging protocol with defined OCT parameters which include a high speed resolution mode, automatic real time (ART) mode ≥20, with a scan pattern of 49 scans, 30°, 120μ separation and centred on the anatomical fovea.

### Assessment of Interobserver Agreement in Determining Features of Disease Activity

**Fig 1:**
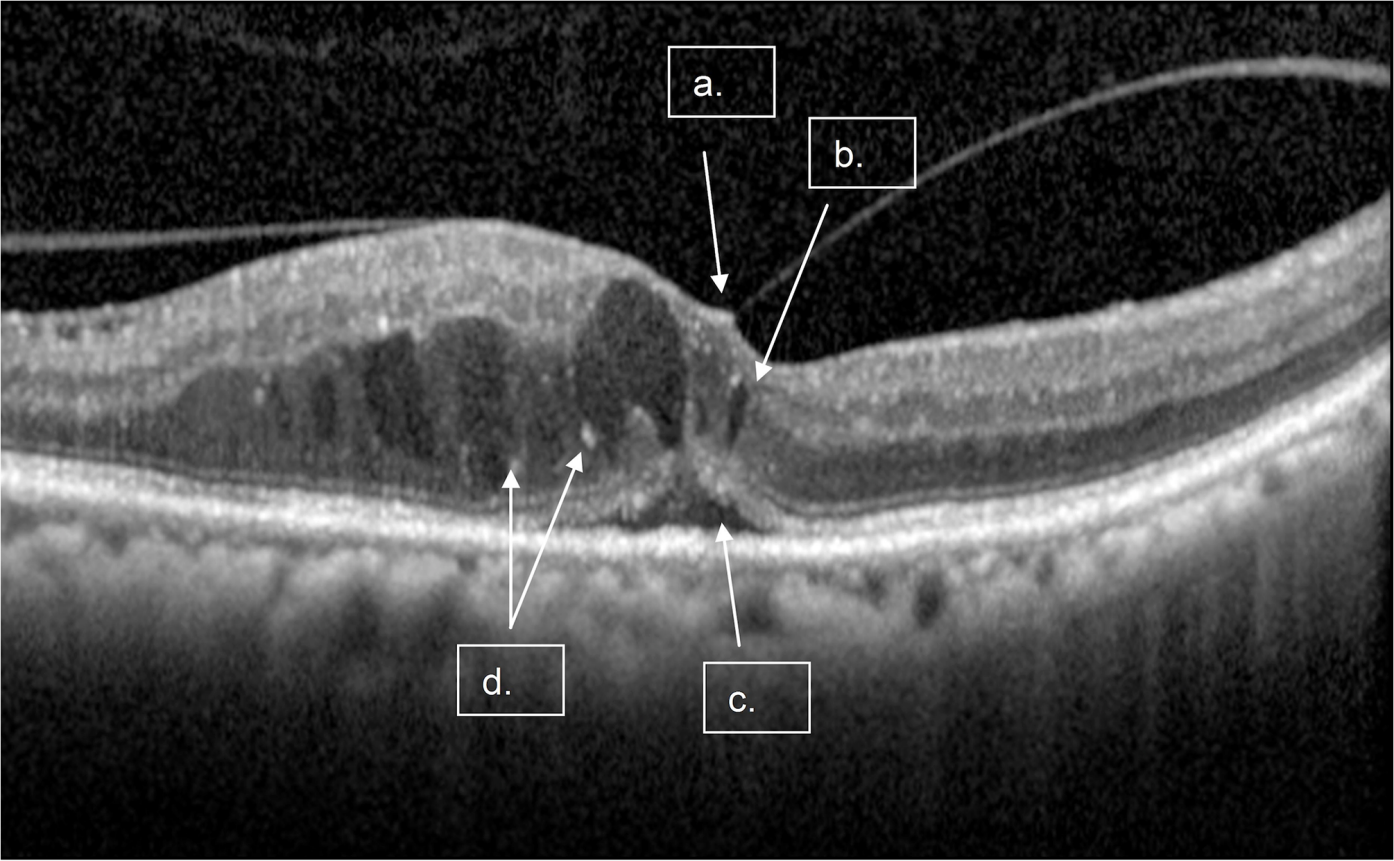
A single scan with macular fluid and Diffuse Retina Edema. Arrows points to the following features: a) Vitreomacular traction (VMT), b) Intraretinal Fluid (IRF); c) Subretinal Fluid (SRF), d) Hyper-reflective Foci (HRF).

Two experienced retinal specialists certified as investigators in retina clinical trials involving OCT-based retreatment decisions (HLZ and MP) independently analysed line scans for the presence or absence of intraretinal fluid (IRF), diffuse retinal oedema (DRE), sub retinal fluid (SRF), macular fluid (MF), hyper-reflective foci (HRF) and vitreomacular traction (VMT) using standardized definitions (Table 1, Fig 1). These definitions were applied in a qualitative manner without reference to standard images. This was a deliberate effort to minimize standardization between the two observers, ensuring the results would be more translatable to clinical practice and investigator-determined retreatment decisions in DME clinical trials.

**Table 1:** Definition of OCT Features of Diabetic Macular Edema (Fig 1).

OCT Feature	Description
IRF	Areas of low reflectivity in the intraretinal space
SRF	Area of low reflectivity in the sub retinal space
DRE	Sponge-like thickening resulting in increased retinal thickness with areas of reduced retinal reflectivity compared with retina without thickening
MF	Either IRF, SRF or DRE present
HRF	Foci of high reflectivity in intra-retinal space
VMT	Hyper-reflective line showing attachment to the inner retinal boundary (vitreo-macular interface) with distortion of the retinal surface

IRF, intraretinal fluid; SRF, subretinal fluid; DRE, diffuse retinal edema; MF, macular fluid; HRF, hyper-reflective foci; VMT, vitreomacular traction

**Fig 2:**
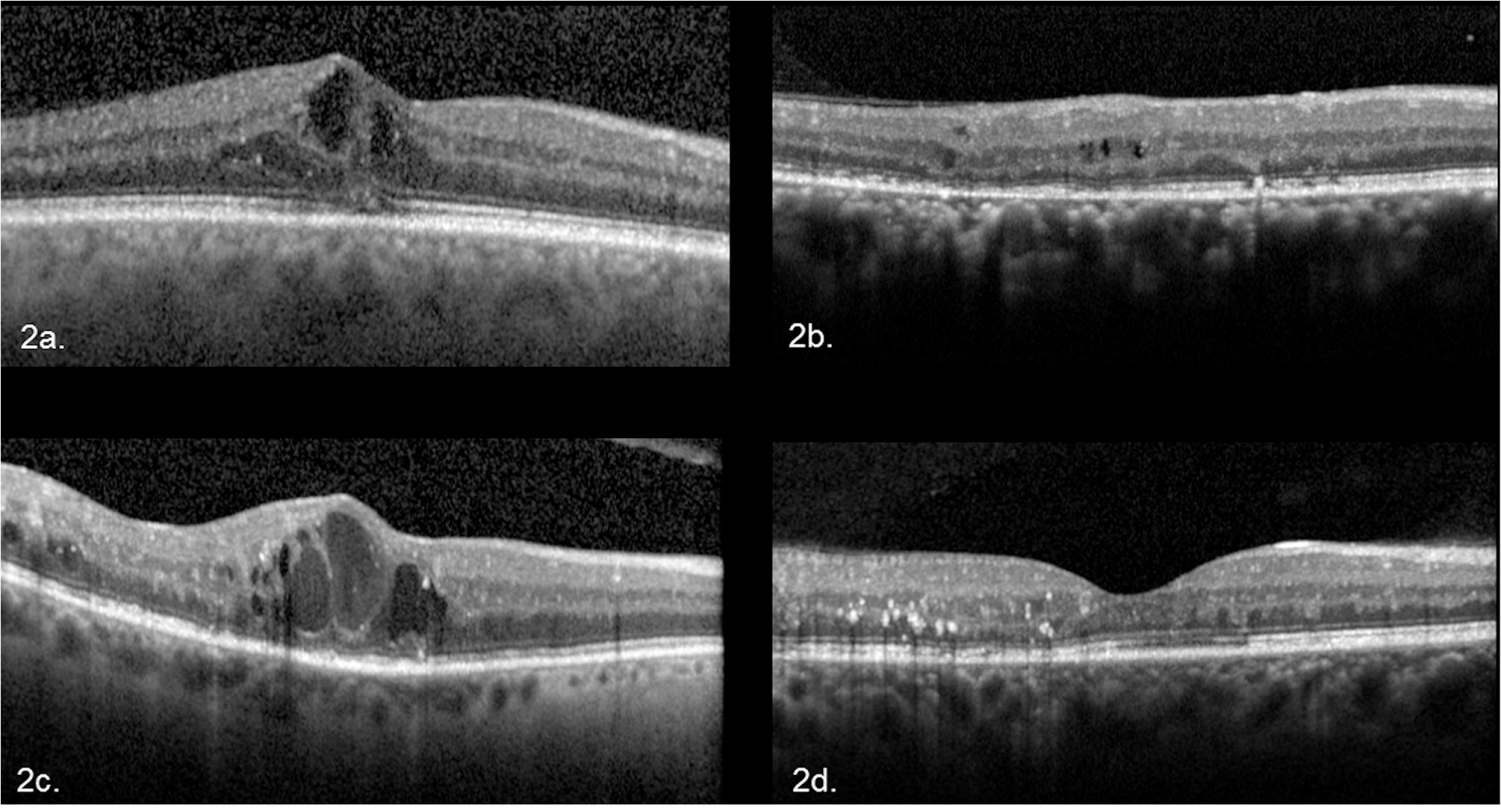
(a-d) Optical coherence tomography images which demonstrate the disparity of assessments between the 2 graders. (a) foveal line scan, with disagreement on presence of Subretinal Fluid (SRF). (b-c) scans from 1mm zone which demonstrates disagreement on presence or absence of Diffuse Retinal Edema (DRE). Both graders agreed Macular Fluid [1] was present. (d) fovea line scan which graders disagreed on presence or absence of Macular Fluid.

Analysis was carried out for the foveal line scan (defined as the scan with the deepest depression in the centre of the scan, or otherwise scan number 25, the middle scan, if depression not present) and for a central 1 mm zone defined by 4 line scans above and 4 line scans below the foveal line scan) and for the whole volume scan (all 49 line scans included in the analysis). In all, 59 scans were analysed per patient and a total of 2950 line scans were analysed this series. Fig 2D demonstrates an example of a single foveal line scan with the deepest depression in the centre of the scan. Scans were analysed by viewing images on a standard computer monitor display using Heidelberg Explorer software version 5.7.4 with Spectralis OCT images database with no further image processing.

The prevalence of each feature (presence of the feature by either observers) was reported. Paired gradings from the two observers were compared using cross-tabulations, percentages of agreement/disagreement, and the kappa statistic (κ, a measure of concordance adjusting for chance agreement). The κ statistic was interpreted in line with the ranges suggested by Landis and Koch <0, poor agreement; 0 to 0.20, slight agreement; 0.21 to 0.40, fair agreement; 0.41 to 0.60, moderate agreement; 0.61 to 0.80, substantial agreement; and >0.80, almost perfect agreement. For features with extremely low or high prevalence, the κ statistic is unstable and difficult to interpret. Analysis was performed by presence of absence of feature in the different pre-defined zones as mentioned above. Analysis was performed on a per eye basis by combining observations made on line scans from each eye for the central 1 mm area and the whole scan however results for the foveal scan represented analysis of a single line scan.

## Results

In total SDOCT scans from 50 eyes undergoing treatment for DME from 39 patients were identified for inclusion in this analysis. There were 6 females and 13 were Caucasian with a mean age (± SD) of 66 years (±10 years). A total of 2950 individual lines scans were analysed and the mean visual acuity using ETDRS (Early Treatment Diabetic Retinopathy Study) letter score was 69 letters (±8 letters) with a mean central macular thickness of 349μm (±75μm). Results are reported for 3 clinically relevant OCT regions: 1 for the foveal line scan 2. For a central 1 mm zone and 3 For the entire volume scan.

The prevalence of SDOCT features of DME detected by both graders is shown in Table 2. There is increasing prevalence of each feature as the number of line scans analysed increased.

**Table 2:** Percentage Agreement and Kappa (κ) Statistic for Each SDOCT Feature of DME for the Foveal Line Scan.

Feature	Percentage Concordance and Kappa	
Feature	%	Κ
IRF	96	0.811
DRE	88	0.737
SRF	96	0.648
MF	100	1.00
HRF	88	0.440
VMT	94	0.765

Percentage concordance and kappa values at the fovea, 1mm zone and entire scan analysis are shown in Tables 2, 3 and 4 respectively. Examples of disagreement are shown in Fig 2. The percentage concordance for SRF remained the same at fovea, 1mm zone and whole scan (96%) and kappa values were substantial (k = 0.648) for SRF in all scans analysed. For analysis of IRF, the percentage concordance was 96%at fovea scans and increased to 98% in the 1mm-zone and whole scan analysis, with substantial to almost perfect kappa values at all 3 regions analysed.

**Table 3:** Percentage Agreement and Kappa (κ) Statistic for Each SDOCT Feature of DME for the Central 1mm zone.

Feature	Percentage Concordance and Kappa	
Feature	%	Κ
IRF	98	0.658
DRE	94	0.879
SRF	96	0.648
MF	98	0.658
HRF	96	0.485
VMT	90	0.767

**Table 4:** Percentage Agreement and Kappa (κ) Statistic for Each SDOCT Feature of DME for the Entire Scan.

Feature	Percentage Concordance and Kappa	
Feature	%	Κ
IRF	98	0.658
DRE	96	0.919
SRF	96	0.648
MF	98	0.658
HRF	96	0.485
VMT	90	0.767

The percentage concordance for DRE increased from 88% at fovea to 94% at 1mm zone scans and 96% for all scans analysed, whilst the percentage concordance for HRF increased from 88% at fovea to 94% at 1mm zones and all scans.

Percentage concordance decreased for MF from 100% at fovea to 98% at 1mm zone scans and whole scans with corresponding kappa values of perfect and substantial kappa values respectively (k = 1.00 and k = 0.658). A decrease in percentage concordance was also noted for VMT from 94% at fovea to 90% at 1mm zone and whole scans, kappa values remained as substantial (k = 0.765 and k = 0.767 respectively.)

## Discussion

The introduction of OCT imaging into clinical practice has seen rapid and unprecedented changes in the way we assess patients with macular disease. The arrival of effective treatments delivered through intravitreous injections to treat macular disease has paralleled these advances in retinal imaging. In an effort to reduce the treatment burden for patients, treatment paradigms with anti-angiogenic agents such as ranibizumab involve an initiation phase of treatment but then further intravitreous injections are given based on signs of disease activity or progression. One of the key determinants of disease activity is the presence of morphological abnormalities of the retina detected on OCT imaging. As the need for further intravitreous injections can often depend on the interpretation of OCT scans, it is important to understand and explore inter-observer agreement in the detection of morphological abnormalities in OCT scans in eyes with DME undergoing treatment. Variability in the detection of macular fluid on OCT imaging can lead to variability in retreatment decisions and therefore lead to variability in treatment outcomes in clinical practice.

In this work we report high rates of agreement in the detection of OCT based morphological abnormalities associated with DME in patients undergoing treatment using SDOCT (Spectralis OCT). Furthermore, agreement for the detection of abnormalities is increased when findings from a larger number of OCT line scans (sampling a larger area of the macular) are pooled or summed. Of the six OCT features evaluated, it was noted that HRF had a low kappa and comparatively poor concordance in all the scans analysed. A reason accounting for this could be due to poor differentiation between artefact and true hyper-reflective foci, especially in cases where there may be minimal macular fluid (Fig 2D). DRE was also noted to have a poor concordance at a single foveal line scan, but increased with larger number of scans. This could be due to difficulties in recognizing ‘spongiform’ type patterns of fluid versus cystic patterns using only a single scan. Based on the results, it appears that IRF, SRF and MF are the most consistent features recognized across different type and number of scans analysed.

In the results, it was also noted that for HRF in all types of scans analysed (foveal, 1 mm, and whole volume scans), there was high inter-grader reliability but only slight agreement in kappa values (Tables 2, 3 and 4). This discrepancy has been noted and commented on in other work and is thought to arise because of the dependence of the k statistic on the prevalence of the feature being studied: very high or low prevalence of a particular feature may therefore give rise to a low k value despite a high percentage concordance[7–9].

Recent clinical trials using intravitreous ranibizumab or bevacizumab for the treatment of DME have relied on non-continuous dosing using OCT imaging to detect signs of DME. These OCT features of morphological abnormalities include IRF, SRF and DRE.[10,11]. In the DRCR study, READ, RESOLVE, BOLT studies, after the initiation phase, the retreatment criteria are driven by VA and/or OCT detection of progression of disease. For instance, the DRCR.net protocol I study based retreatment criteria on investigator’s interpretation of morphological abnormalities on OCT images. This may lead to variability in retreatment decisions, potentially compromising long-term vision outcomes.[12] To the best of our knowledge, despite these OCT based retreatment paradigms there have been no studies reporting the interobserver agreement in interpreting SDOCT based morphological abnormalities in DME. In previous work, we reported good but not perfect agreement in detecting morphological abnormalities of the retina in time-domain OCT scans of eyes receiving treatment for neovascular age-related macular degeneration[13]. Several other studies have also found good reproducibility and repeatability of OCT derived macular thickness measurements in eyes with DME using the Spectralis OCT [14–16].

The strengths of this study include the large number of OCT line scans analyzed and that the study was carried out in a clinical setting. Though this latter aspect of the study could be perceived as a weakness when compared to interobserver agreement studies in a reading center, the results from our study are more generalizable to clinical practice. The weakness of our study is that eyes were at different stages of treatment and it may be beneficial to consider interpreting scans from eyes which had received a standardized amount of treatment or follow-up (eg analyzing scans from eyes receiving one year of follow-up and treatment)

In summary, the results of this study suggest that there is good agreement between observers when interpreting retinal morphological abnormalities in OCT imaging at both single line scans and combination of multiple scans in eyes of patients receiving treatment for DME. As OCT imaging is used to help determine the need for retreatment with pharmcotherapies such as ranibizumab, it is important to confirm that interpretation of OCT features of DME disease activity is not subject to significant interobserver variability.
