# Correction: Interobserver Agreement in Detecting Spectral-Domain Optical Coherence Tomography Features of Diabetic Macular Edema

**DOI:** 10.1371/journal.pone.0142023

**Published:** 2015-10-29

**Authors:** Ling Zhi Heng, Maria Pefkianaki, Philip Hykin, Praveen J. Patel

The second author’s name is incorrectly spelled. The correct name is Maria Pefkianaki. The correct citation is: Heng LZ, Pefkianaki M, Hykin P, Patel PJ (2015) Interobserver Agreement in Detecting Spectral-Domain Optical Coherence Tomography Features of Diabetic Macular Edema. PLoS ONE 10(5): e0126557. doi:10.1371/journal.pone.0126557

## References

[1] Heng LZ, Pefianaki M, Hykin P, Patel PJ (2015) Interobserver Agreement in Detecting Spectral-Domain Optical Coherence Tomography Features of Diabetic Macular Edema. PLoS ONE 10(5): e0126557 doi:10.1371/journal.pone.0126557 25996150 10.1371/journal.pone.0126557 PMC4440774

